# Combination therapy of cisplatin with cilastatin enables an increased dose of cisplatin, enhancing its antitumor effect by suppression of nephrotoxicity

**DOI:** 10.1038/s41598-020-80853-6

**Published:** 2021-01-12

**Authors:** Masashi Arita, Satoshi Watanabe, Nobumasa Aoki, Shoji Kuwahara, Ryo Suzuki, Sawako Goto, Yuko Abe, Miho Takahashi, Miyuki Sato, Satoshi Hokari, Aya Ohtsubo, Satoshi Shoji, Koichiro Nozaki, Kosuke Ichikawa, Rie Kondo, Masachika Hayashi, Yasuyoshi Ohshima, Hideyuki Kabasawa, Michihiro Hosojima, Toshiyuki Koya, Akihiko Saito, Toshiaki Kikuchi

**Affiliations:** 1 Department of Respiratory Medicine and Infectious Diseases, Niigata University Graduate School of Medical and Dental Sciences, 1-757 Asahimachidori, Chuouku, Niigata, 951-8510 Japan; 2 Laboratory of Clinical Nutrition, Department of Nutrition, Graduate School of Human Cultures, The University of Shiga Prefecture, Hikone, Japan; 3 Department of Applied Molecular Medicine, Kidney Research Center, Niigata University Graduate School of Medical and Dental Sciences, Niigata, Japan; 4 Department of Clinical Nutrition Science, Niigata University Graduate School of Medical and Dental Sciences, Niigata, Japan

**Keywords:** Chemotherapy, Toxicology

## Abstract

Cisplatin, one of the most active anticancer agents, is widely used in standard chemotherapy for various cancers. Cisplatin is more poorly tolerated than other chemotherapeutic drugs, and the main dose-limiting toxicity of cisplatin is its nephrotoxicity, which is dose-dependent. Although less toxic methods of cisplatin administration have been established, cisplatin-induced nephrotoxicity remains an unsolved problem. Megalin is an endocytic receptor expressed at the apical membrane of proximal tubules. We previously demonstrated that nephrotoxic drugs, including cisplatin, are reabsorbed through megalin and cause proximal tubular cell injury. We further found that cilastatin blocked the binding of cisplatin to megalin in vitro. In this study, we investigated whether cilastatin could reduce cisplatin-induced nephrotoxicity without influencing the antitumor effects of cisplatin. Nephrotoxicity was decreased or absent in mice treated with cisplatin and cilastatin, as determined by kidney injury molecule-1 staining and the blood urea nitrogen content. Combined with cilastatin, a twofold dose of cisplatin was used to successfully treat the mice, which enhanced the antitumor effects of cisplatin but reduced its nephrotoxicity. These findings suggest that we can increase the dose of cisplatin when combined with cilastatin and improve the outcome of cancer patients.

## Introduction

Cis-dichlorodiammineplatinum [II] (cisplatin) is one of the most active cytotoxic agents in the treatment of cancers, such as lung cancer, head and neck cancer, esophageal cancer, gastric cancer, colorectal cancer, urothelial cancer, bladder cancer and testicular cancer. Furthermore, recent evidence has demonstrated that combination treatment with immune-checkpoint inhibitors and platinum-doublet chemotherapy, including cisplatin, has promising antitumor effects against various malignancies^1,2^. Although cisplatin has been shown to play an important role in the treatment of cancers, cisplatin is more poorly tolerated than other chemotherapeutic drugs. The main dose-limiting toxicity of cisplatin is its nephrotoxicity, which is dose-dependent^3^. Impairment of renal function is a clinical problem in 20–35% of patients who receive a cisplatin-containing regimen^4^. Although previous studies reported less toxic methods of cisplatin administration, such as hydration with supplemental magnesium and mannitol, we showed that acute kidney injury was still observed in 21.4% of patients with thoracic malignancies treated with cisplatin^5^.

Megalin, a large (approximately 600 kD) glycoprotein member of the LDL receptor family, is expressed at the apical membrane of proximal tubular epithelial cells (PTECs)^6,7^. Megalin mediates intracellular signal transduction and plays a pivotal role in the reabsorption of diverse glomerular-filtered substances^8^. Nephrotoxic drugs resorbed by megalin accumulate in PTECs and induce their apoptosis^9^. We previously demonstrated that cisplatin, vancomycin and colistin are reabsorbed via megalin and induce PTEC injury^10^.

Cilastatin is used as a compounded drug with imipenem and prevents the nephrotoxicity of imipenem by competing with renal dehydrodipeptidase I (DHP-I), which is located in the brush border of PTECs, reducing the accumulation of imipenem^11^. Our previous study demonstrated that cilastatin binds megalin and attenuates the interaction between megalin and nephrotoxic drugs, including cisplatin, vancomycin and colistin, in vitro^10^. Coadministration of colistin and cilastatin resulted in reduced tubular damage in mice.

The maximum tolerated dose of cisplatin, unlike other anticancer agents, is determined by cisplatin-induced nephrotoxicity. Here, we investigated whether cilastatin effectively interferes with the binding of cisplatin to megalin and suppresses cisplatin-induced nephrotoxicity without reducing the antitumor effects of cisplatin. We also evaluated whether coadministration of cilastatin would enable an increased dose of cisplatin and augment the antitumor effects of cisplatin.

## Results

### Cilastatin suppresses cisplatin nephrotoxicity

**Figure 1:**
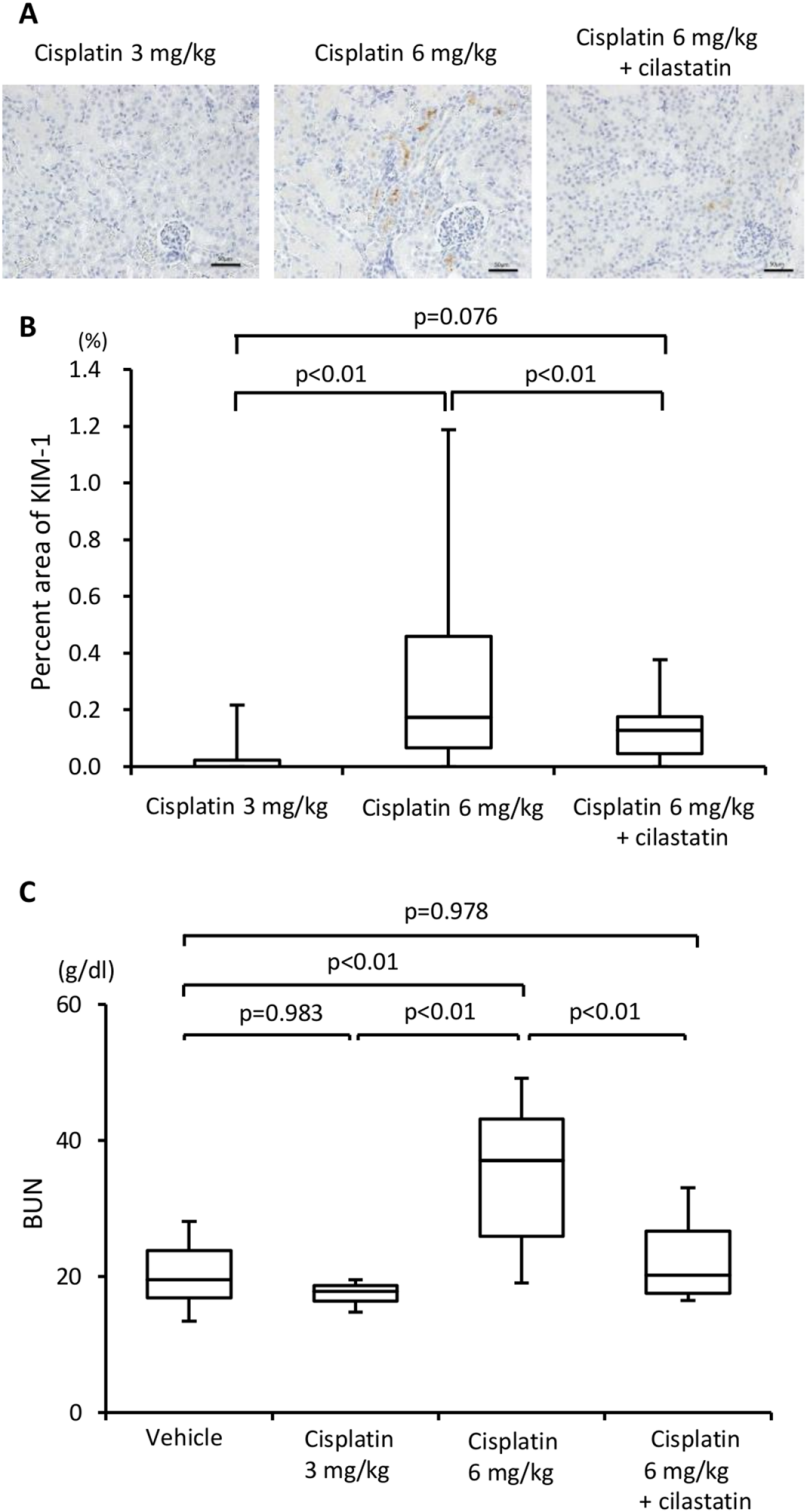
Administration of cilastatin suppressed cisplatin-induced kidney injury. (**A**) BALB/cA mice were administered cisplatin i.p. at a dose of 3 mg/kg or 6 mg/kg on days 0, 3 and 6. Mice that received 6 mg/kg cisplatin were treated s.c. with cilastatin (100 mg/kg) from days 0 to 6 or left untreated. Nine days after the first administration of cisplatin, the kidneys were harvested and stained with KIM-1. (**B**) The expression of KIM-1 was scored to evaluate the percentage of injured tubules. The KIM-1 staining scores were lower in mice treated with cisplatin with cilastatin combination therapy. (**C**) BUN was measured by enzymatic methods.

Previously, we demonstrated that cisplatin bound megalin in vitro and that cisplatin nephrotoxicity was reduced in megalin-knockout proximal tubular cells^10^. In the current study, we investigated whether administration of cilastatin would suppress cisplatin-induced PTEC injury, the major pathologic feature induced by cisplatin. To explorer the maximum tolerated dose of cisplatin, mice were administered with cisplatin at several doses. Although 3 mg/kg cisplatin did not increase the expression of KIM-1, an established kidney injury marker, 6 mg/kg cisplatin augmented the KIM-1-positive area in PTECs (Fig. 1A,B). These results indicated that cisplatin at a dose of 6 mg/kg exceeded the maximum tolerated dose. Coadministration of cilastatin with cisplatin significantly reduced the expression of KIM-1 on PTECs. Because cisplatin nephrotoxicity was determined by elevated serum markers, such as creatinine and blood urea nitrogen (BUN), in cancer patients, we assessed BUN in mice receiving cisplatin with or without cilastatin. As shown in Fig. 1C, a significant increase in BUN was observed in mice treated with 6 mg/kg cisplatin. In contrast, cilastatin significantly decreased the BUN level. We further evaluated whether administration of cilastatin would suppress other adverse events. Coadministration of cilastatin with 6 mg/kg cisplatin did not affect the number of white blood cells or red blood cells in the peripheral blood (supplementary Fig. 1A,B). Additionally, body weight loss due to cisplatin was not affected by cilastatin (supplementary Fig. 1C).

### Megalin is not expressed on human and mouse tumor cells

**Figure 2:**
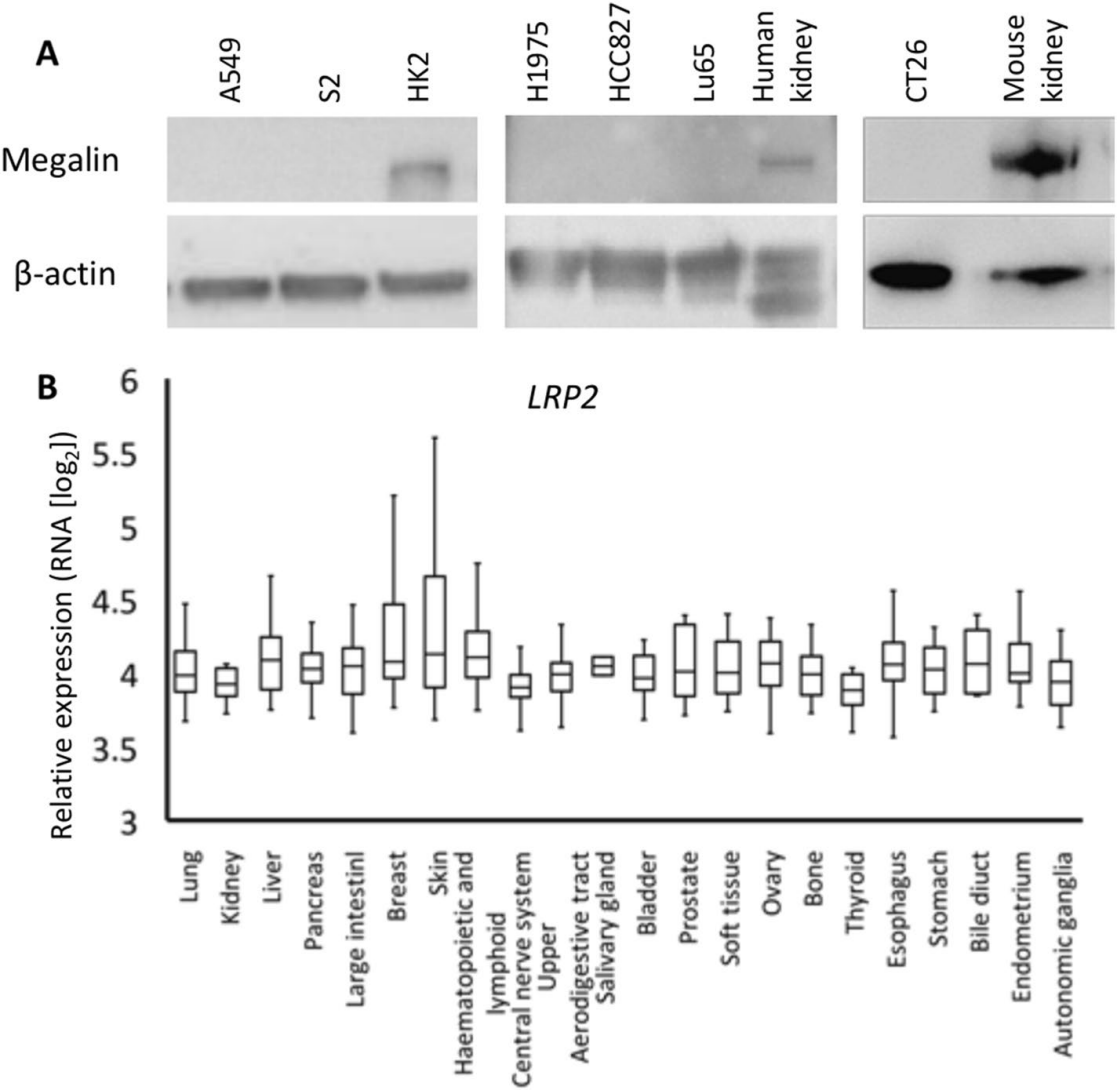
Megalin is not expressed in cancer cell lines. (**A**) Megalin expression in human lung cancer cell lines, a mouse colon adenocarcinoma cell line, human kidney lysate and mouse kidney lysate was determined by western blotting. The representative plots of 3 different experiments are shown. (**B**) mRNA expression of *LRP2* (encoding megalin) in various cancers from the CCLE database. Normalized expression from the microarray data was calculated by robust multichip analysis (RMA).

Megalin may be expressed on tumor cells and play a role in cisplatin uptake by tumor cells. Thus, we evaluated the expression of megalin on mouse CT26 tumor cells and human lung cancer cell lines by western blotting. As shown in Figs. 2A and S2, we found that none of the cancer cell lines expressed the megalin protein. We further assessed whether other human cancer cells express megalin by investigating microarray data from the Cancer Cell Line Encyclopedia (CCLE)^12^. This approach revealed that the median expression levels of *LRP2*, which encodes megalin, in cancers originating from different tissues were similar to those in lung cancers (Fig. 2B).

### Cilastatin does not influence the antitumor effects of cisplatin

**Figure 3:**
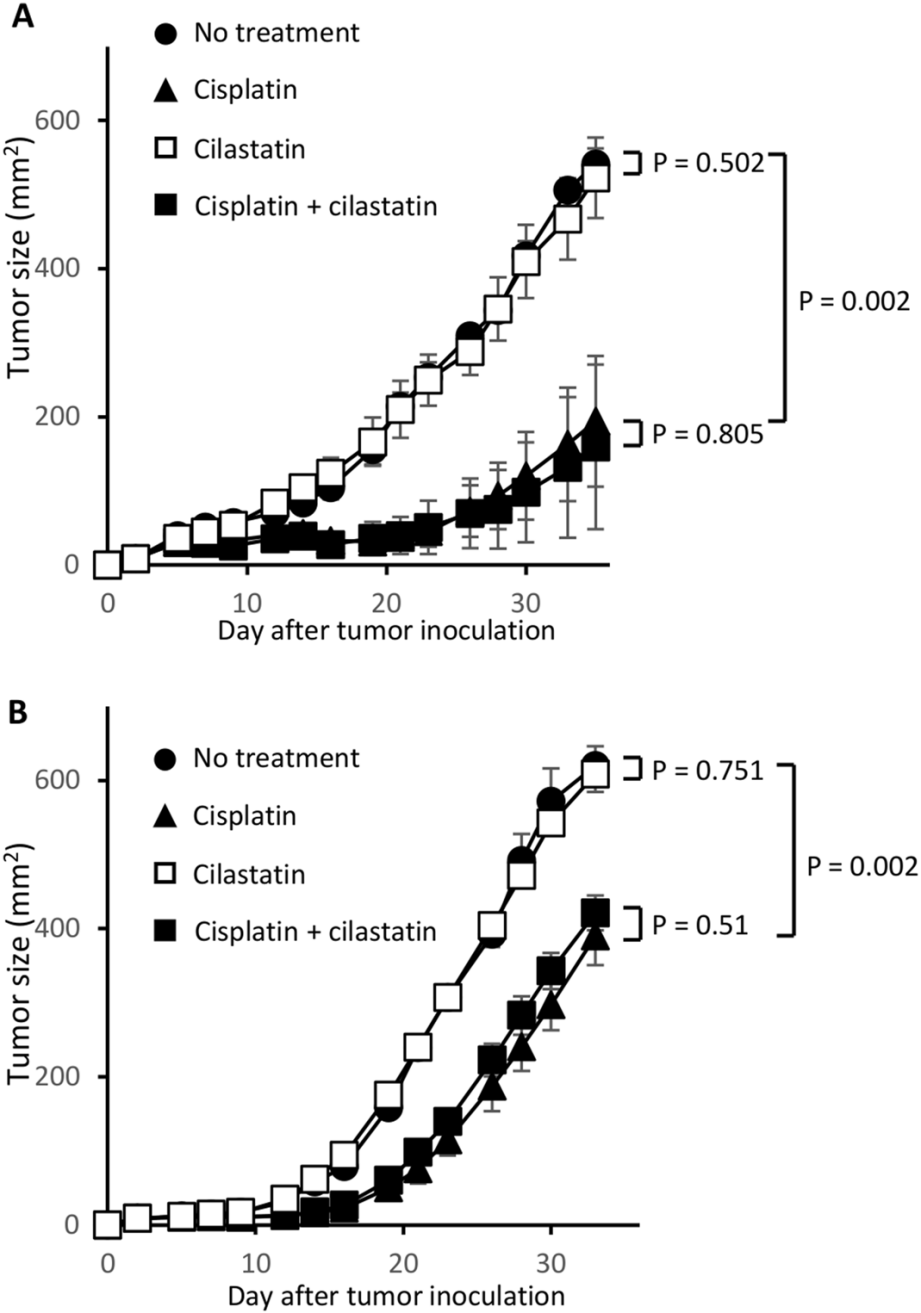
Influence of cilastatin on the antitumor effects of cisplatin. (**A**) BALB/cA mice were inoculated s.c. with CT26 tumor cells along the midline of the abdomen. On days 2 and 5, the mice were treated i.p. with cisplatin (4 mg/kg) with or without administration of cilastatin (100 mg/kg) s.c. from days 2 to 8. The resultant skin tumors were measured in 2 perpendicular directions 3 times per week, and the tumor areas (mm^2^) were recorded. (**B**) SCID mice were injected s.c. with A549 tumor cells. The mice were administered cisplatin (3 mg/kg) i.p. on days 2, 5 and 8 with or without treatment with cilastatin (100 mg/kg) s.c.

We next asked whether the blockade of megalin by cilastatin treatment would affect the antitumor effects of cisplatin in vivo. BALB/c mice were inoculated s.c. with CT26 mouse colon adenocarcinoma cells. To evaluate whether cilastatin influences the antitumor effects of cisplatin in human cancer cells, SCID mice were injected with A549 human lung adenocarcinoma cells. The mice were treated with 4 mg/kg cisplatin 2 times or 3 mg/kg cisplatin 3 times. The amount of cisplatin used in this experiment was determined such that it did not cause cisplatin-induced nephrotoxicity in normal mice. From the start date of cisplatin treatment, cilastatin was administered once daily for 7 days. As shown in Fig. 3A,B, coadministration of cilastatin and cisplatin did not decrease the antitumor effects of cisplatin.

### Cisplatin with cilastatin augments the antitumor effects of cisplatin by increasing the amount of cisplatin

**Figure 4:**
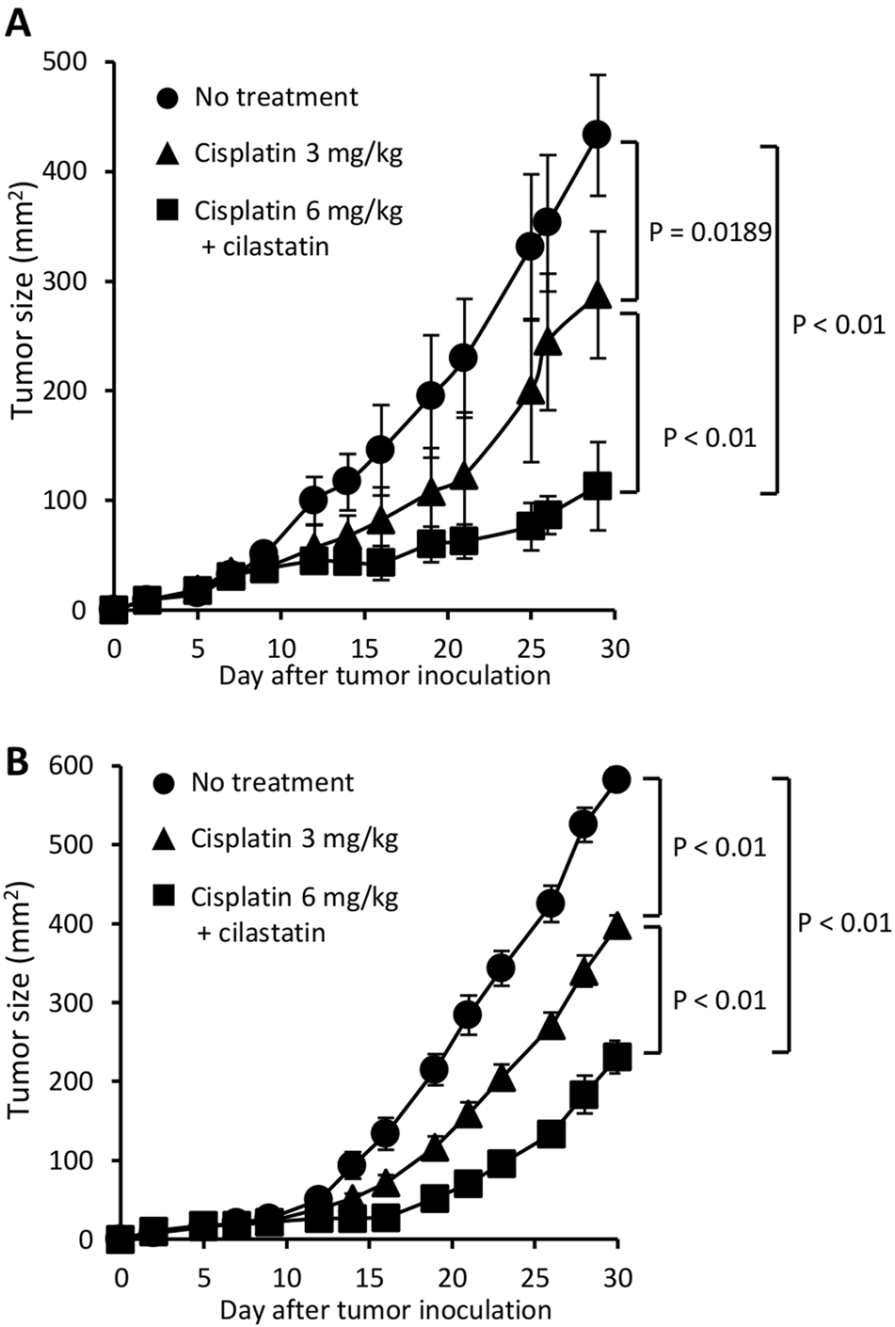
Antitumor effects of cisplatin combined with cilastatin. BALB/cA mice were inoculated s.c. with CT26 tumor cells (**A**), and SCID mice were injected s.c. with A549 tumor cells (**B**). The mice were treated with 3 mg/kg or 6 mg/kg cisplatin i.p. on days 6, 9 and 12 with or without administration of cilastatin s.c. from days 6 to 12.

Because the dose-limiting toxicity and maximum tolerated dose of cisplatin were determined by renal impairment, suppression of cisplatin nephrotoxicity by cilastatin may allow us to safely increase the dose of cisplatin and improve the outcome of tumor-bearing hosts. To test this hypothesis, skin tumor-bearing mice were treated with cisplatin at a dose of 3 mg/kg or 6 mg/kg. As shown in Fig. 1A–C, 6 mg/kg cisplatin caused significantly PTEC injury and increased BUN; however, 3 mg/kg cisplatin and the combination of 6 mg/kg cisplatin and cilastatin did not induce renal impairment. The combination of 6 mg/kg cisplatin and cilastatin significantly reduced human and mouse tumor progression (Fig. 4A,B).

### Cisplatin, carboplatin, oxaliplatin and pemetrexed are bound by megalin, which is competitively inhibited by cilastatin

**Figure 5:**
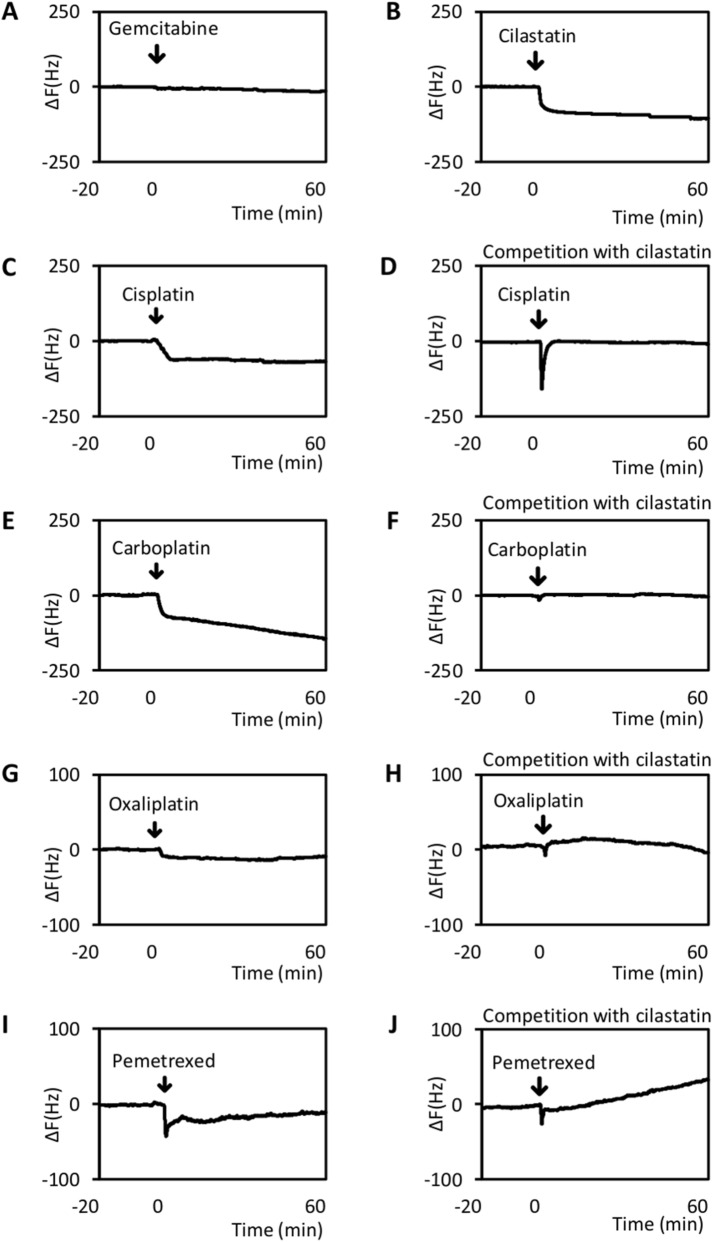
QCM analysis. (**A**) Gemcitabine (20 mg/ml), (**B**,**D**,**F**,**H**,**J**) cilastatin (10 mg/ml), (**C** and **D**) cisplatin (1.38 mg/ml), (**E** and **F**) carboplatin (4.44 mg/ml), (**G** and **H**) oxaliplatin (7 mg/ml) and (**I** and **J**) pemetrexed (2.85 mg/ml) in buffer B were administered at the time points indicated by the arrows. Megalin was immobilized on the QCM sensor chip to evaluate the binding of anticancer drugs to megalin. QCM analysis revealed that gemcitabine did not bind megalin (**A**); however, megalin was bound by cilastatin (**B**), cisplatin (**C**), carboplatin (**E**), oxaliplatin (**G**) and pemetrexed (**I**). Cilastatin competed with these drugs for megalin binding (**D**, **F**, **H** and **J**).

In addition to cisplatin, other anticancer agents were reported to cause renal impairment^13^. To investigate whether other anticancer agents also bind megalin and whether cilastatin competes with anticancer drugs other than cisplatin for megalin binding, we performed QCM analysis. Cisplatin, carboplatin, oxaliplatin and pemetrexed bound megalin immobilized on the sensor chip; however, gemcitabine did not interact with megalin (Fig. 5A,C,E,G,I).

Next, we used QCM analysis to determine whether the interactions between these drugs and megalin were inhibited by cilastatin. Similar to cisplatin, cilastatin competed with these anticancer agents for megalin binding (Fig. 5B,D,F,H,J).

## Discussion

Cisplatin has severe nonhematological toxicities, particularly nephrotoxicity and gastrointestinal toxicity. The recent development of new antiemetic therapies has greatly reduced cisplatin-induced nausea and vomiting; however, cisplatin-induced nephrotoxicity remains an unsolved problem. We and others have reported that 20–35% of cancer patients receiving cisplatin develop renal impairment even with toxicity-reducing methods of cisplatin administration^4,5^. Although the mechanisms of cisplatin nephrotoxicity are not fully understood, several factors were indicated to be involved in cisplatin nephrotoxicity. Intracellular delivery of cisplatin in tubular cells induces activation of apoptotic signals and inflammation, resulting in renal tubular cell death. Organic cation transporters (OCTs) are highly expressed in the proximal and distal tubules of the kidneys. These transporters mediate cisplatin uptake from the basolateral side into renal tubular cells^14^. We previously demonstrated that megalin is located at the brush border membranes of PTECs and mediates the reabsorption of cisplatin^6,7,10^. Megalin-deficient PTECs were protected from injury in kidney-specific mosaic megalin-knockout mice treated with cisplatin^10^. In the current study, we demonstrated that cilastatin blocks the binding of cisplatin to megalin and significantly suppresses cisplatin-induced nephrotoxicity in vivo. Because the dose-limiting toxicity of cisplatin is renal impairment, a reduction in cisplatin nephrotoxicity may allow us to increase the dose of cisplatin and enhance the antitumor effects of cisplatin. Indeed, we successfully treated mice with cisplatin at an increased dose without PTEC injury when we concurrently administered cilastatin and cisplatin. Significant suppression of tumor growth was observed in mice treated with the combination of cilastatin and cisplatin at the higher dose.

Previous studies have reported the mechanism of cisplatin transport. Ishida et al. demonstrated that the copper transporter protein Ctr1 mediates cisplatin uptake and that a reduction in the Ctr1 protein decreased the intracellular accumulation of cisplatin^15^. The copper efflux transporters ATP7A and ATP7B mediate cisplatin efflux, and overexpression of ATP7A during platinum-based chemotherapy was associated with poor survival in ovarian cancer patients^16,17^. To exclude the possibility that megalin is expressed on cancer cells and associated with the antitumor effects of cisplatin, we evaluated megalin expression on cancer cell lines using western blot analysis and investigated microarray data from the CCLE. None of the human lung cancer cell lines examined expressed megalin, and similar mRNA expression levels of megalin were observed in lung cancers and in cancers originating from different sites in the microarray data. Coadministration of cilastatin and cisplatin did not decrease the antitumor effects of cisplatin in a tumor-bearing mouse model. Although these findings indicate that blockade of megalin does not suppress the antitumor effects of cisplatin, the influence of cilastatin on the antitumor effects of cisplatin should be further assessed in clinical settings.

As mentioned above, cisplatin is one of the most active anticancer agents; however, cisplatin use is avoided in patients with chronic kidney disease (CKD) due to the risk of deterioration of renal functions. Combination therapy of cisplatin with cilastatin might allow us to use cisplatin for cancer patients with CKD. In addition to cisplatin, other anticancer agents cause renal impairment^13^. The results of our QCM analysis revealed that carboplatin, oxaliplatin and pemetrexed also have the ability to bind megalin. Because cilastatin competed with these anticancer drugs for megalin binding, cilastatin could suppress their nephrotoxicity. Our previous study demonstrated that 95.2% of patients with thoracic malignancies have at least one risk factor for CKD^5^. These patients have a higher risk for the development of renal insufficiency after chemotherapy. Although platinum plus pemetrexed chemotherapy is the standard of care for advanced nonsquamous non-small-cell lung cancer (non-sq NSCLC) patients and mesothelioma patients, real-world data showed that 17.3% of NSCLC patients discontinued pemetrexed therapy due to renal impairment^18^. Administration of cilastatin could prevent renal impairment and enable these patients to continue platinum plus pemetrexed chemotherapy. Recent evidence has shown that the addition of PD-1/PD-L1 blockade therapy to platinum-based chemotherapy prolonged overall survival in advanced NSCLC patients, SCLC patients and head and neck cancer patients^1,2,19–21^. PD-1/PD-L1 blockade therapy was reported to cause nephritis and increase the risk of renal impairment^1,22^. Further studies to assess whether cilastatin administration reduces the nephrotoxicity of chemoimmunotherapy are warranted.

In conclusion, our data indicated that cilastatin blocks the interaction of cisplatin and megalin and reduces cisplatin-induced nephrotoxicity without influencing the antitumor effects of cisplatin. The combination therapy of cisplatin with cilastatin enables an increase in the dose of cisplatin, enhancing its antitumor effect by suppressing nephrotoxicity. Furthermore, cilastatin competed with other anticancer drugs including carboplatin, oxaliplatin and pemetrexed for megalin binding. These findings suggest that cilastatin could suppress their nephrotoxicity. Because there is a clear dose–response relationship between tumor regression and chemotherapy, the combination therapy of anticancer agents and cilastatin may allow us to increase the dose of these anticancer agents and improve survival in cancer patients. We are now conducting a phase I study to assess the safety and pharmacokinetics of combination therapy of cilastatin with cisplatin in NSCLC patients (UMIN000030363).

## Methods

### Mouse

Female C57BL/6J, BALB/cA and C.B-17/Icr-scid mice (severe combined immune deficiency, SCID) were purchased from CLEA Laboratory (Tokyo, Japan). The mice were housed in a specific pathogen-free environment and used at the age of 8 to 12 weeks. The experimental protocols were on the basis of the National Institutes of Health Guide for the Care and Use of Laboratory Animals and approved by the Niigata University Institutional Animal Care and Use Committee.

### Chemicals and cell lines

Cisplatin was generously supplied by Bristol-Myers Squibb (New York, NY, USA). Gemcitabine, carboplatin, oxaliplatin, pemetrexed and cilastatin were purchased from Sigma-Aldrich (St. Louis, MO, USA).

CT26 colon adenocarcinoma cells from BALB/c mice, HK-2 human proximal tubule epithelial cells, and the A549, HCC827 and H1975 human pulmonary adenocarcinoma cell lines were obtained from the American Type Culture Collection (Manassas, VA, USA). S2 and Lu85 human pulmonary small-cell carcinoma cells were kindly gifted by Dr. S. Shu (Cleveland Clinic, OH, USA). All cell lines were cultured in RPMI-1640 medium supplemented with 10% heat-inactivated fetal bovine serum (FBS) and 10% CO_2_ at 37 °C. Whole human kidney lysate was obtained from Novus Biologicals (Littleton, CO, USA).

### Competition of cilastatin for cisplatin-induced nephrotoxicity

BALB/cA mice were treated intraperitoneally (i.p.) with cisplatin at the indicated doses on days 0, 3 and 6. Cilastatin (100 mg/kg) was administered subcutaneously (s.c.) once daily from days 0 to 6. At day 9, the mice were anesthetized via intraperitoneal injection of pentobarbital and sacrificed. The left kidneys were dissected and processed for semiquantitative morphological analysis and kidney injury molecule-1 (KIM-1) immunostaining.

### Immunohistochemistry for KIM-1

Kidney tissues were stained with KIM-1 as previously described^10^. Briefly, horizontal cross-sections containing the hila of the kidneys were obtained and fixed in 4% paraformaldehyde phosphate buffer for 72 h at room temperature. After fixation, the tissues were dehydrated in a graded ethanol series from 70 to 100%, cleared in xylene, and embedded in paraffin. From each tissue sample, 4 μm-thick sections were cut using a microtome (REM-710; Yamato Kohki Industrial Co., Ltd., Asaka, Japan). To detect KIM-1, the avidin–biotin–peroxidase complex method was used. Goat anti-mouse KIM-1 antibody (1:700; AF1817; R&D Systems, Minneapolis, MN, USA) was used as the primary antibody. Kidney tissue sections were deparaffinized by immersion in xylene and rehydrated through a graded ethanol series. Antigen retrieval was accomplished by heating the slides in a microwave in 10 mM citrate buffer, pH 6.0. Sections were incubated in 3% H_2_O_2_ for 20 min at room temperature. After incubation with 5% rabbit serum in PBS to block nonspecific binding, endogenous biotin binding was eliminated with an Avidin/Biotin Blocking Kit (Vector Laboratories, Burlingame, CA, USA). Finally, immunostaining signals were developed with the DAB Substrate-Chromogen System (DAKO, Carpinteria, CA, USA), and the sections were counterstained with Mayer hematoxylin. Negative control staining with PBS instead of the primary antibody was always performed in parallel.

### Histology analysis of nephrotoxicity

Images of five cortical regions (400× magnification) containing no glomeruli from KIM-1-stained kidney sections of BALB/cA mice treated with cisplatin were randomly taken using a BZ-8000 microscope (Keyence, Osaka, Japan)^23^. The images were used to determine the percent area of KIM-1-positive cells using Image-Pro Plus v.7.0. The mean values for each mouse were statistically evaluated.

### Western blot analysis

Protein expression levels were determined by western blot analysis. Briefly, CT26, A549, S2, H1975, HCC827 and Lu85 cells were lysed with 10% sodium dodecyl sulfate. Mouse kidney, human kidney and HK-2 cell lysates were used as positive controls for western blotting. The cell lysates were centrifuged, and the supernatants were collected to measure the amount of protein using BCA protein assay reagent (Thermo Scientific, Waltham, MA, USA). Equal amounts of total protein from each sample were subjected to sodium dodecyl sulfate–polyacrylamide gel electrophoresis on Mini-PROTEAN TGX Any kD Precast Gels (Bio-Rad, Hercules, CA, USA), after which the proteins were transferred to polyvinylidene difluoride membranes (Millipore, Billerica, MA, USA) and immunostained with primary antibodies against megalin^24^ and mouse β-actin (AC-15, Abcam, England). The secondary antibody was horseradish peroxidase (HRP)-conjugated goat anti-mouse IgG (Bio-Rad, CA, USA). HRP was detected using the ECL western detection kit (Pierce, Rockford, IL, USA).

### Mouse tumor model

BALB/cA mice were s.c. inoculated with 1.5 × 10^6^ CT26 mouse colon adenocarcinoma cells along the midline of the abdomen. To establish xenograft model, C.B-17/Icr-scid mice were s.c. injected with 2.5 × 10^6^ A549 human lung adenocarcinoma cells along the midline of the abdomen. On days 2, 5, and 8, the mice were injected i.p. with cisplatin (3 to 6 mg/kg) with or without subcutaneous administration of cilastatin (100 mg/kg) from days 2 to 8. The tumor sizes in two perpendicular dimensions were measured three times per week with digital calipers, and the tumor areas (mm^2^) were recorded.

### Quartz crystal microbalance (QCM) analysis

Binding of the drugs to megalin was determined using an AFFINIX Q8 instrument (Initium, Kanagawa, Japan) as previously described^10^. Megalin was prepared from rat kidneys by immunoaffinity chromatography as previously described^25^ and immobilized onto QCM sensor tips using an immobilization kit for AFFINIX (ULVAC, Chigasaki, Japan) as previously described^26,27^. The sensor tips were soaked in buffer B (10 mM HEPES, pH 7.4, 150 mM NaCl, and 2 mM CaCl_2_) in the incubation chamber. Anticancer drugs were injected into the chamber, and the change in resonance frequency was recorded and used to evaluate binding of the drugs to megalin. Each drug was dissolved in 8 ml of buffer B to its maximum concentration. Cilastatin was used to compete with the other drugs for megalin binding. Cilastatin at 10 mg/ml was first added to the chamber, and after its binding to megalin had reached a steady state, each of the drugs was injected into the chamber to assess competitive binding inhibition by cilastatin. The resonance frequency of the QCM at equilibrium was defined as the zero position. Both the stability and drift of the 27 MHz QCM frequency in solution were 3 Hz.

### Statistical analysis

For experiments in the animal tumor model, the significance of differences between groups was analyzed using Student’s t-test. All other experimental data were analyzed by one-way ANOVA, and each group was compared by Bonferroni’s multiple comparison test. All statistical analyses were carried out using GraphPad Prism 6 (GraphPad Software, Inc., La Jolla, CA, USA) and EZR (Saitama Medical Center, Jichi Medical University, Saitama, Japan)^28^. A 2-tailed P value of < 0.05 indicated statistical significance. All experiments were repeated at least twice.

## Supplementary Information

Supplementary Information.

## Data Availability

The data generated or analyzed during the current study are available from the corresponding authors on reasonable request.
