# Supplementary material for: Combination therapy of cisplatin with cilastatin enables an increased dose of cisplatin, enhancing its antitumor effect by suppression of nephrotoxicity
Source: Sci Rep. 2021 Jan 12;11:750. doi: 10.1038/s41598-020-80853-6 (PMC7804437; doi:10.1038/s41598-020-80853-6)
Supplement: Supplementary file 1 — Supplementary Information. [file 41598_2020_80853_MOESM1_ESM.pdf]

Supplementary Information

Combination therapy of cisplatin with cilastatin enables an increased dose of cisplatin, enhancing its antitumor effect by suppression of nephrotoxicity

Masashi Arita[1], Satoshi Watanabe[1*], Nobumasa Aoki[1], Shoji Kuwahara[2], Ryo Suzuki[1], Sawako Goto[3], Yuko Abe[1], Miho Takahashi[1], Miyuki Sato[1], Satoshi Hokari[1], Aya Otsubo[1], Satoshi Shoji[1], Koichiro Nozaki[1], Kosuke Ichikawa[1], Rie Kondo[1], Masachika Hayashi[1], Yasuyoshi Ohshima[1], Hideyuki Kabasawa[4], Michihiro Hosojima[4], Toshiyuki Koya[1], Akihiko Saito[3] and Toshiaki Kikuchi[1]

[1] Department of Respiratory Medicine and Infectious Diseases, Niigata University Graduate School of Medical and Dental Sciences

[2] Laboratory of Clinical Nutrition, Department of Nutrition, Graduate School of Human Cultures, The University of Shiga Prefecture

[3] Department of Applied Molecular Medicine, Kidney Research Center, Niigata University Graduate School of Medical and Dental Sciences

[4] Department of Clinical Nutrition Science, Niigata University Graduate School of Medical and Dental Sciences

Supplementary Figure 1.

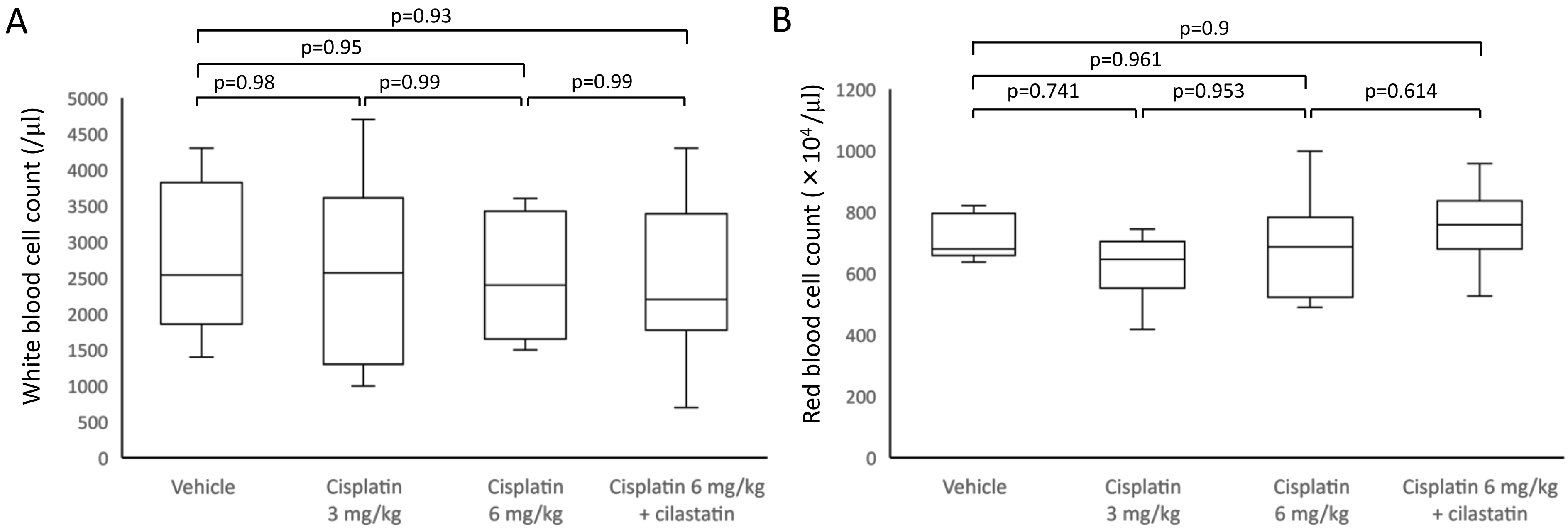

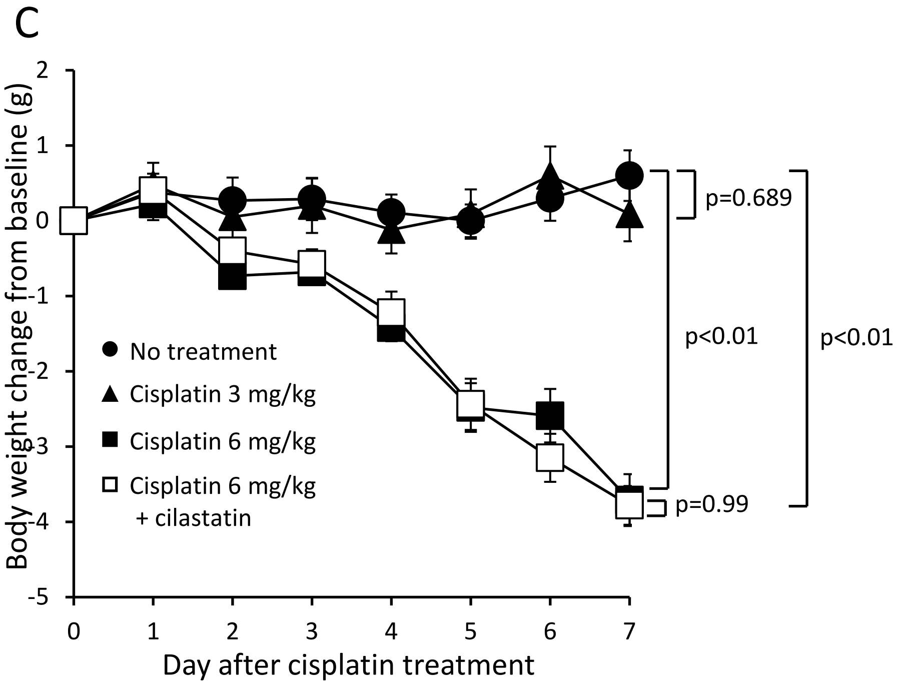

Figure S1: Cilastatin did not reduce the adverse events of cisplatin other than renal toxicity.

BALB/cA mice were treated with 3 mg/kg or 6 mg/kg cisplatin i.p. on days 0, 3 and 6. The mice were treated with cilastatin s.c. or left untreated from days 0 to 6. On day 14, white blood cells (A) and red blood cells (B) in the peripheral blood were counted.

(C) BALB/cA mice were treated with 3 mg/kg or 6 mg/kg cisplatin i.p. on days 0, 3 and 6 with or without cilastatin administration. Changes in body weight from baseline were measured once daily.

Supplementary Figure 2.

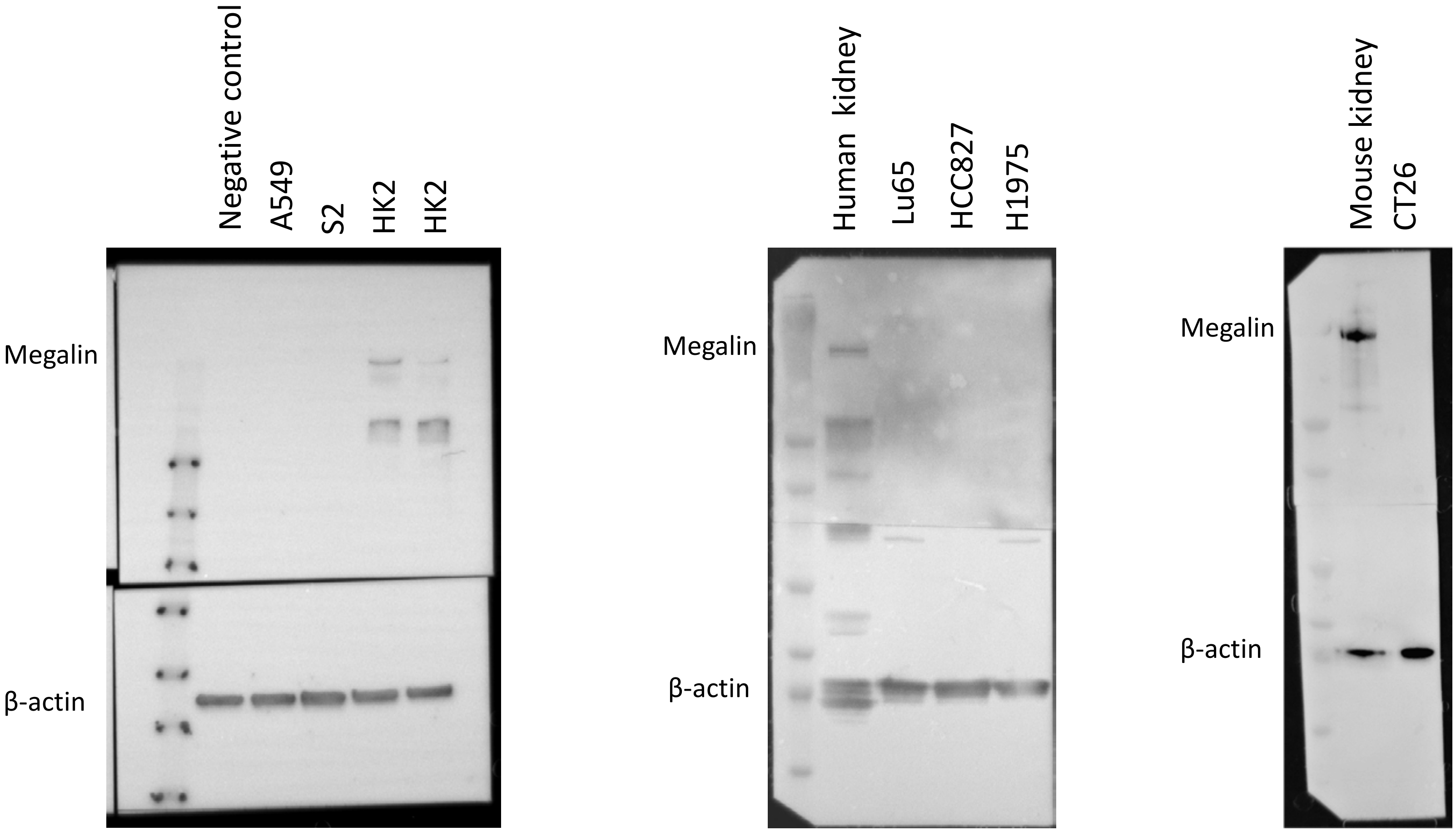

Figure S2: Megalin is not expressed in cancer cell lines.

Megalin expression in human lung cancer cell lines (A549, S2, Lu65, HCC827 and H1975), proximal tubule epithelial cell line (HK2), a mouse colon adenocarcinoma cell line (CT26, human kidney lysate and mouse kidney lysate was determined by western blotting.
